# Genetic Regulation and Breeding Application of Medium-Chain Fatty Acids Metabolism in Rice

**DOI:** 10.3390/biology14121674

**Published:** 2025-11-25

**Authors:** Muhammad Zubair, Xiaohong Tong, Aneela Ashraf, Hongzhou Li, Guanghao Li, Ai Xin, Jiale Chen, Yifeng Wang, Zhiyong Li, Jie Huang, Yu Cheng, Jian Zhang, Jiezheng Ying

**Affiliations:** 1 State Key Laboratory of Rice Biology and Breeding, China National Rice Research Institute, Hangzhou 311400, China; zubairbiochem1334@gmail.com (M.Z.); tongxiaohong@caas.cn (X.T.); m18064079352@163.com (H.L.); guanghao_12@163.com (G.L.); aixin0127@outlook.com (A.X.); c18572321185@outlook.com (J.C.); wangyifeng@caas.cn (Y.W.); lizhiyong@caas.cn (Z.L.); huangjie@caas.cn (J.H.); chengyu@caas.cn (Y.C.); 2 Institute of Urban Agriculture, Chengdu Agricultural Science and Technology Centre, Chinese Academy of Agricultural Sciences, Chengdu 610000, China; aneelaashraf43@gmail.com

**Keywords:** rice (*Oryza sativa* L.), medium chain fatty acids, metabolism, regulatory mechanism, crop breeding

## Abstract

Medium-chain fatty acids (MCFAs), with carbon chain length of 6 to 12 carbon atoms, have received substantial attention in metabolism and applications in health, agriculture and industry. They have shown promising therapeutic effects for metabolic disorders such as obesity, diabetes, and neuro-triglycerides (MTGs) enriched in fatty acids. Rice (*Oryza sativa* L.) is one of the most widely used starchy crops around the world. New varieties have a lower level of starch and include lipid profiles that could make rice oil possess better nutritional quality and generate novel possibilities in biofuel production. With the help of new genetic and breeding techniques, the expression of genes that involve fatty acid biosynthesis can be altered. CRISPR/Cas9 and marker-assisted selection (MAS) are currently enhancing the fatty acid content in rice varieties without affecting the yield and various agronomic traits. This review presents the progress on the knowledge of rice MCFA biosynthetic pathways, genetic determinants of MCFA biosynthesis, and breeding technologies for enhancing MCFA production in rice. It also addresses wider implications of this research, including possible enhancement in human nutritional quality and development of sustainable agriculture.

## Simple Summary

Rice is an important food but contains a low content of healthy medium-chain fatty acids (MCFAs), special fats found in certain foods, including coconut oil, which have been shown to provide various health benefits. Here, we review progress made in efforts to flavor rice with these beneficial MCFAs. Through advanced breeding and genetic engineering, researchers can alter how rice produces oils to develop new varieties with more valuable nutrients. If that were to be carried out, the new enriched rice would offer people healthier food choices and, at the same time, produce raw materials that could be sustainably used to make biofuels and industrial chemicals. This type of research may lead to the expansion of rice beyond a cereal to a more useful crop for communities.

## 1. Introduction

Medium-chain fatty acids (MCFAs), classified by a carbon chain length of 6–12 atoms, are a class of fatty acids that exhibit distinct metabolic and physiological effects compared to long-chain fatty acids [1]. This makes MCFAs ideal for both nutritional and industrial purposes. Due to their shorter carbon chains compared to long-chain fatty acids (LCFA), MCFAs are absorbed more rapidly in the gastrointestinal tract. However, they are also directly metabolized in the liver, providing a quick source of energy [2]. At room temperature, the even-chain fatty acids with nine or fewer carbon atoms are liquid. The lower members of this group tend to have a strong, unpleasant odor. Lipophilicity of MCFAs, as a measure of the free acid content, was generally comparable to that of the LCFAs. Because of their lower lipophilicity than LCFAs, SCFAs do not form micelles and are not part of biological membranes [3]. Their alkali metal salts are therefore extensively hydrolyzed in water. Members of the water-soluble family are believed to have a high propensity to form bimolecular associates when in an aqueous solution [4].

Consequently, MCFAs have a potential impact on clinical treatments that include neurological diseases, metabolic disorders, and weight management through the ketogenic diet [5]. Beyond human health, MCFAs also hold promise in agriculture, particularly in enhancing crop oil composition and contributing to biofuel production. Moreover, the peroxisomes are equipped with carnitine-acetyltransferase and carnitine-octanoyl transferases, which convert shortened acyl-CoAs into the carnitine esters to be exported to the mitochondria [6]. In pathological conditions, such as inborn deficiency of medium-chain acyl-CoA dehydrogenase, octanoate and deaconate can accumulate to significant concentrations in tissues, leading to a dysfunction of the mitochondrial respiratory complexes [7]. These disorders are characterized by increased urinary excretion of dicarboxylic MCFAs, mainly adipic, suberin, and sebacic acids, which originate from the microsomal oxidation of the corresponding MCF acyl-CoAs. Emulsions containing these triglycerides were administered to patients with impaired digestion or absorption of normal LCFA-containing triglycerides [8].

The metabolism of fatty acids in plants is an important mechanism that supports plant life functions and provides an important energy source for humans. In human body, certain important fatty acids cannot be synthesized, the consumption of plant oils derived from seeds has become a primary source of these fatty acids [9]. Plant seeds typically store fatty acids in the form of triacylglycerol (TAGs), which are made of a glycerol molecule bonded to three fatty acids. These oils act as the principal energy reserves in plant seeds, providing essential energy for early seedling development and germination [10]. Increasingly, research is focusing on enhancing the quantity and types of FAs in rice to meet dietary and industrial demands. Major goals in crop breeding are the selection of novel genotypes with higher fatty acid content and a better proportion of unsaturated fatty acids [11].

**Figure 1:**
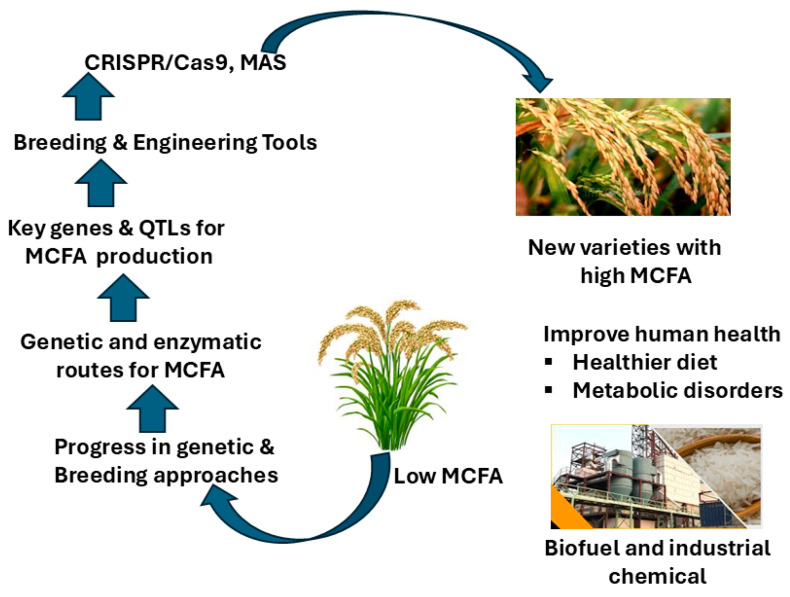
Summary of the integration of the strategies for MCFA increase in rice. Figure shows the deployment of breeding and engineering tools such as CRISPR/Cas9, and marker-assisted selection (MAS), to target important genes and QTLs for MCFA biosynthesis. These new lines have the potential to be used for biofuel and bio-based products, as they overcome the natural low MCFA content that currently limits rice development by altering both genetic and enzymatic pathways.

Metabolic processes responsible for lipid biosynthesis in plants are complicated. Transitory accumulation of the synthesized sucrose in the developing seed is subsequently hydrolyzed to glucose and fructose during late seed development [12]. Rice (*Oryza sativa* L.) is a staple food worldwide, normally high in starch but low in oil content especially in fatty acids. It was previously known that rice oil is a low value byproduct, rich in long-chain fatty acids [13]. Higher fat content in rice, especially MCFA content, has been achieved through breeding and genomic engineer technologies, including CRISPR/Cas9 and MAS [14]. These approaches target genes involved in fatty acid metabolism or their intermediates, thereby enhancing the metabolic flow toward the production of FAs while ensuring that other agronomically important traits, e.g., drought tolerance or disease resistance and yield, are not adversely affected. In plants, lipids in the seed are primarily located in the embryos and aleurone layers, where they are stored as TAG within oil bodies [15] (Figure 1).

In young mammals, primarily human infants, mothers’ milk is an important source of MCFAs, which are primarily present as triglycerides and phospholipids. For example, MCFAs (C7:0–C12:0) contribute 6–17% and 9–28% fatty acids in bovine and human milk, respectively. MCFAs can also be synthesized in mammalian and human tissues, especially liver. Rice bran, a byproduct of milling, is rich in oil and is used to produce edible oil. On a dry weight basis, oil content in rice bran ranges from 17.3% to 27.4%, while that of brown rice ranges from 2.75% to 4.49% [16]. Despite this, quantitative genetic methods have been used to identify QTLs associated brown rice oil content [17]. This review summarizes the current understanding of MCFA metabolism in rice, with a main focus on the genetic determinants of MCFA biosynthesis and the application of breeding techniques to enhance MCFA content in rice germplasm. In addition, we discuss the implications of these advancements for human health, sustainability in agriculture, and biofuel production, highlighting the potential societal significance of this emerging field of study.

## 2. Fatty Acid Synthesis Mechanism

Fatty acids are the major form of oil, and their biosynthesis takes place mainly from plastids. Biosynthesis of FA starts when acetyl-CoA carboxylase (ACCase) produces malonyl-CoA (Mal-CoA) from acetyl-CoA, and the subsequent elongation of carbon chains is started with the help of fatty acid synthase (FAS) system via a series of reactions that involve condensation, reduction, dehydration, and re-reduction [18]. The FAS system is a multienzyme complex comprising of at least 5 enzymes, with an acyl carrier protein (ACP) involved and all catalysis occurs on ACP. This system enables an iterative process of chain elongation, in which two carbon units are added in each cycle [19]. The unsaturated FAs are synthesized by the influence of the desaturating enzymes, which produce monounsaturated FAs, such as oleic acid, palmitoleic acid and long-chain polyunsaturated fatty acids resulting from linolenic acid and linoleic acid [20]. FATB functions as the master regulator of saturated FA chain length. The substrate preference determines whether a plant is an MCFA or an LCFA producer. For example, C14:0 and C16:0 are the main products of elongation by *MYR2*- and *PAL6*-encoded FATB enzymes in traditional rice lines [21]. In the case of coconut (*Cocos nucifera*), an MCFA-rich species, specific *FATB* isoforms have high activity toward C8:0–12:0 acyl-ACPs (e.g., *CnFATB1*) [22]. Ectopic overexpression of such specialized *FATBs* is capable of rewiring the lipid profile at a primary level and redirecting carbon flux from LCFA to MCFA. Other plant species can use the well-characterized genetic structures and pathways involved in storage oil production in *Arabidopsis* and maize as a Model [23]. By integrating data from those species with present findings, a streamlined mechanism for fatty acid production in rice can be proposed [24]. During the elongation cycle, malonyl-ACP supplies two-carbon units, while acetyl-coA (CoA) serves as the initial substrate. Carbon chain elongation is catalyzed by 3-ketoacyl-ACP synthases *KASI*, *KASII*, and *KASIII*, which act on intermediates of different chain lengths [25]. Of these elongases, KASII is a secondary node of control that affects the supply of substrate to FATB. By diverting elongation of C16:0-ACP to C18:0-ACP, KASII activity pulls carbon away from the medium-chain fatty acid pool for LCFA synthesis [26]. Thus, a decrease in *KASII* expression would lead to an accumulation of C16:0-ACP. This works in synergy with a tailored MCFA-specific FATB, which resulting in a higher overall yield of shorter-chain fatty acids by providing more substrate for chain termination [27].

In plastids, C18:0 is dehydrogenated to C18:1, and OsFAD2 then dehydrogenates it in the endoplasmic reticulum to generate C18:2 [28]. As a substrate for OsFAD2 in the synthesis of linoleic acid (C18:2), LIN6, a PDCT, transfers C18:1 to the phosphatidylcholine (PC) pool. OsFAD3 then acts on this substrate to produce C18:3 [29]. The elongation process of long-chain FA is facilitated by 3-ketoacyl-CoA synthase ARA6. The genes and QTL identified show an important advancement in understanding the molecular processes governing fatty acid biosynthesis in rice [30].

**Figure 2:**
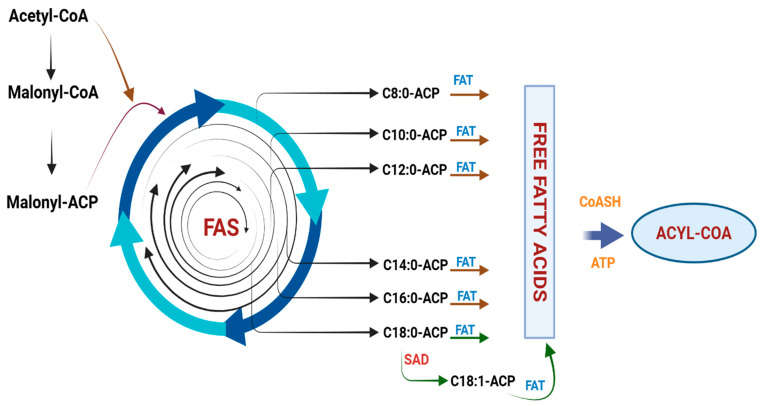
Process of fatty acid synthesis with the help of fatty acid thioesterase (FAT). Export of FAs with the help of fatty acid thioesterase type B (FATB) is represented by red arrows. Green arrows show the hydrolysis of C18:1 by fatty acid thioesterase type B (FATB). Furthermore, free FAs are converted into acyl-CoAs with the help of Coenzyme A in due to adenosine triphosphate (ATP). SAD, steric acid desaturase; FAS, fatty acid synthase; ACP, acyl carrier protein; FAT, fatty acid thioesterase.

Numerous important lipid metabolism genes have been cloned and their functions thoroughly examined by mutation analysis in earlier *Arabidopsis* research [31]. This has led to the development of two comprehensive databases of *Arabidopsis* lipid genes with better annotation [32]. QTL analysis was carried out for a series of fatty acids (14:0, 16:0, 18:0, 18:1, 18:2, 18:3, 20:0) which are important metabolites in the FA biosynthesis process. This approach enables the discovery of critical enzymes in lipid metabolism and the characterization of the associated metabolic pathways [33]. Variation in the phenotypes controlled by the target QTLs coincided with the roles of the candidate genes [34]. A gene putatively encoding an acyl-ACP thioesterase (FatB) was mapped near QTL pal6 at the top of chromosome 6. FatB catalyzes the hydrolysis of acyl–ACPs to form free fatty acids and ACP, which is highly specific for the chain length of 16:0. The acyltransferases are required to synthesize the storage lipids as they transfer the acyl chain from the CoA-ester to the glycerol backbone [35] (Figure 2).

## 3. Triacylglycerol (TAG) Biosynthesis in Plants

The condensation of MCFAs to form TAG is a challenging bottleneck and an opportunity in engineering quality-improved rice. There are multiple possible routes, but the Kennedy pathway is the major de novo TAG biosynthesis route [36]. In order for MCFAs to be retained in rice oil, their incorporation into the glycerol backbone has to be effective, which is controlled by substrate specificity of crucial acyltransferases, serving as “gate keepers” in neutral lipid metabolism [37]. The first committed step is catalyzed by the enzyme sn-1 glycerol-3-phosphate acyltransferase (GPAT) which transfers a fatty acid to the glycerol-3-phosphate (G3P), forming lysophosphatidic acid (LPA) [38]. This esterification reaction is the first step in the biosynthesis of various glycerolipids, including membrane lipids and TAG, and is crucial for cellular metabolism [39]. In *Arabidopsis thaliana*, GPAT9 (*AtGPAT9*) which is homologous to mammalian GPATs was identified as the sn-1 GPAT in vitro [40]. Suppression of *AtGPAT9* expression reduces LPA and TAG synthesis, and its knockout is embryo-lethal, indicating a required role for this gene in plant growth [41]. The second reaction of the Kennedy pathway, which is the addition of a second fatty acid to the sn-2 position of LPA to produce PA, is catalyzed by lysophosphatidic acid acyltransferase (LPAAT) [42]. This enzyme is essential for plant embryogenesis and its mutation leads to embryonic lethality [43]. LPAAT enzymes from different plants have been characterized, particularly those found in coconut, palm, meadowfoam, and *Cuphea* species [44]. Importantly, the substrate specificity of LPAAT defines which fatty acid is esterified at the sn-2 position of TAG, a decisive branch point in oil synthesis. Most of the widely used LPAATs from major crops such as regular rice, the preference for Long-Chain Acyl-CoA (C16 and C18) was strongly confirmed, which creates a bottleneck in fatty acid metabolism if MCFA are introduced [45]. However, LPAATs from species rich in MCFA-containing oils, such as coconut and *Cuphea*, have developed a special preference for MCFA-CoA (C10:0, C12:0, and C14:0). Thus, overexpression of these MCFA-preferring LPAATs is a required approach for overcoming such bottlenecks and to achieve efficient transportation of MCFAs into the pathway for TAG synthesis in rice [46]. Overexpression of LPAATs increases metabolic flux through the Kennedy de novo synthesis route and thus TAG levels. LPAATs substrate specificity largely determines the nature of storage and membrane lipids [47].

Several putative genes have been identified in *A. thaliana*, but the candidate for the major PAPs involved in de novo DAG synthesis remain unknown [48]. The last step of TAG biosynthesis, esterification of DAG, is catalyzed by the enzyme diacylglycerol acyltransferase (DGAT), which uses the sn-3 position in order to attach a third fatty acid, and the product of this reaction now becomes TAG [18]. There are several non-redundant DGAT isoforms in plants. The two enzymes, DGAT1 and DGAT2, catalyse the same reaction but have different functions and phylogenetic origins. DGAT1 is considered as a major enzyme in the mass secretion of TAG in seeds, and it associates with the ER membrane. In contrast, DGAT2s have a more restricted role, often with significant specificity to unusual and/or specific fatty acid substrates [49]. For example, in plants such as castor bean and tung tree, DGAT2 is highly expressed during seed development and directly involved in the esterification of ricinoleic acid and eleostearic acid to TAG, respectively. This functional divergence further indicates that DGAT2 would be a better target for metabolic engineering of the incorporation of novel fatty acids, such as MCFAs in rice [50].

The substrate preferences of DGAT1 have been investigated to better understand its effect on oil structure. In rapeseed (*Brassica napus*), DGAT1 shows higher specificity for C18:1 as an acceptor in TAG biosynthesis [51]. Acyltransferase activity carried by EaDAcT provides a new example of the substrates that can be accepted by members of the DGAT family other than long-chain acyl-CoAs in TAG biosynthesis, illustrating the diversity of both structural and functional features among the members of this protein family. DGAT, the enzyme responsible for catalyzing the terminal and committed step of TAG biosynthesis, is another important regulatory point [46]. Although LCFAs tend to be its preferred native substrates, it is intrinsically promiscuous with the promise that isoforms discovered or engineered with a higher affinity for MCFA-rich DAG substrates will lead to increased recovery of the final pool of MCFA-enriched TAGs in rice [52].

In addition, to the Kennedy pathway, other routes participate in TAG synthesis and turnover. Phospholipid: diacylglycerol acyltransferase (PDAT) contributes, yet it accepts an acyl group directly from the sn-2 of a PL to form TAG. The presence of this enzyme becomes especially important for channelling less abundant fatty acid-derived TAGs and might easily constitute a bypass route when DGAT activity is limited [53]. In addition, Lysophosphatidylcholine Acyltransferase (LPCAT) is needed in the editing cycle of fatty acids on phosphatidylcholine (PC). The reversible acyl transfer between PC and the acyl-CoA pool, where desaturation takes place, is catalyzed by LPCAT. This activity may permit PUFAs synthesized on PC to be re-esterified into the acyl-CoA pool and subsequently incorporated into TAG by DGAT or PDAT, thus affecting the ultimate composition of TAG [54].

**Figure 3:**
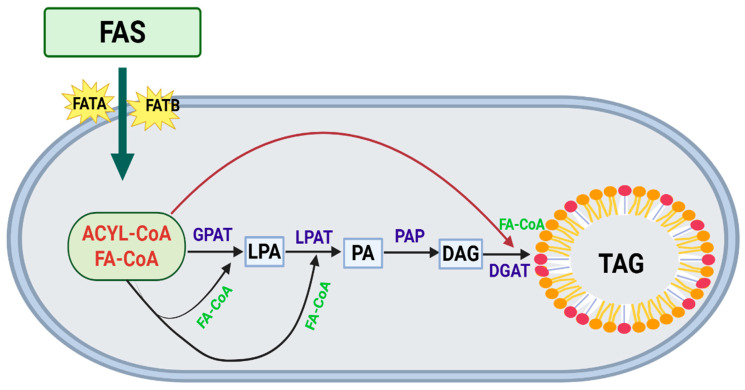
Diagrammatic representation of TAG biosynthesis. FAS, fatty acid synthase; FATA, fatty acid thioesterase type A; FATB, fatty acid thioesterase type B; GPAT, glycerol phosphate acyltransferase; LPAT, lysophosphatidic acid acyltransferase; PAP, phosphatidic acid phosphatase; DGAT, diacylglycerol acyltransferase; LPA, lysophosphatidic acid; PA, phosphatidic acid; DAG, diacylglycerol; TAG, triacylglycerol.

In general, enzyme activities related to TAG synthesis, GPAT, LPAAT, PAP, and DGAT, each provide a significant contribution to both the total amount of TAGs and the FA profiles in seed oils. Knowledge of the substrate preferences of these enzymes is key to the production of tailored plant oils for specific nutritional or industrial applications. [55]. For MCFA-accumulating rice, a combined genetic approach to redirect the carbon flux toward MCFAs through FATB activity, in concert with the expression of compatible MCFA-selective acyltransferases that are such as LPAAT, is necessary to ensure proper routing of these novel fatty acids to store TAGs effectively [56] (Figure 3).

## 4. Regulation of MCFA Metabolism

Improved oil production in several important crops is a primary objective of world agriculture to support the increasing need for this renewable resource [57]. The first attempt to improve oil in plants received relatively little attention. It focused primarily on the TAG assembly pathway, which is commonly characterized by the overexpression of enzymes that convert fatty acids into triacylglycerol [58]. The transcription factor *WRINKIED1* (*WRI1*) plays a key role in controlling seed oil biosynthesis and deposition. *WRI* regulates fatty acid synthesis and glycolysis directly through activating the genes involved in fatty acid synthesis [59]. The *WRI1* gene protein contains two functional AP2/EREB motifs that control the conversion of sucrose to oil. The mutant had 80% less oil and increased sucrose levels, with partial loss of activity of enzymes involved in glycolysis [60]. According to cDNA microarray studies, *WRI* promotes oil biosynthesis primarily by upregulating key enzymes involved in the plastidial glycolysis process during transcription [61].

In *Brassica*, *BnWRI1* overexpression not only enhanced seed oil content 18–38% but also induced early flowering. Also, tissues from *Brachypodium distachyon* grain leaves that were transformed to express *WRI1OL* have up to 32.5-fold higher TAG levels and 2-fold higher free FA content. Upkeeping metabolic homeostasis, therefore involves a fine-tuned balance of synthesis and degradation pathways during fatty acid metabolism [62]. Although *WRINKLED1* (*WRI1*) is a key activator of fatty acid synthesis, its activity is counteracted by peroxisomal β-oxidation, the major degradation pathway in which enzymes such as acyl-CoA oxidase (ACX) and the ABCD1/PXA1 transporter mediate this reaction [63]. Feedback mechanisms, Potentially, the negative feedback that determines TAG synthesis levels could occur through several pathways: (1) Energy sensing by SnRK1 kinase phosphorylation and inactivation of ACCase under energy-limited conditions; (2) Product feedback upon accumulation of acyl-CoAs, such as oleoyl-CoA directly inhibits ACCase activity; (3) Direct metabolic cross-talk where acetyl-CoA and NADPH generated from β-oxidation establish substrate-level inhibition that will fluctuate fatty acid synthase cycle extension; and 4 transcriptional coordination governed by master regulators LEC2 and FUS3 which hierarchically control *WRI1* expression and hepatosynthesis related genes [64]. This functionally integrated network homeostatically maintains metabolism by tuning synthesis rates in a post hoc and product-dependent manner to degradation products and to adaptations in cellular energy status.

Under certain stress conditions or when exposed to defined growth regimes, rice plants may adjust their fatty acid profiles to balance energy storage with membrane fluidity, potentially leading to higher MCFA yield [65].

## 5. Roles of MCFAs on Rice Physiology

Although the core physiological functions of MCFAs in rice remain largely unexplored, they are expected to play important roles in energy storage, membrane structure, and stress tolerance, as demonstrated across various plant systems [66]. The metabolic simplicity of MCFAs, compared to LCFAs, may also benefit rice genotypes grown in high-stress environments, such as drought, high-temperatures, or nutrient deficiencies [67]. Moreover, the synthesis of MCFAs into rice may help to restructure the liposome of the plant for improved plant growth and development (Table 1).

The regulation covers *OsWRI1a* activation of glycolytic (*PK*, *PDH-E1α*) or fatty acid biosynthetic genes *(KASIII*, *ENR1*), with byproducts from the carbon flux being shifted into MCFA production in conjunction with designated thioesterases [62].

New findings from molecular studies on lipid metabolic engineering in rice from CRISPR/Cas9-mediated *OsFAD2-1* knockout reveals a potential homeostasis of the fatty acid profile, balancing oleic acid (more than 40%) with polyunsaturated fatty acids, and field performance without any yield penalty [73]. Meanwhile, overexpression of *OsWRI1a* demonstrates that it is a master regulator of oil biosynthesis by activating both glycolytic (*PK* and *PDH-E1α*) and fatty acid synthesis (*KASIII* and *ENR1*), genes thereby increasing total lipid content in leaves and developing endosperms by 40–65% and >30%, respectively. Constitutive expression of *OsWRI1a*, however, results in severe pleiotropic effects such as reduced growth, leaf chlorosis and low grain weight showing some potential trade-offs for metabolism in carbon partitioning [74]. The tissue-specific expression patterns of *OsFAD2-1* in seeds and *OsWRI1a* in embryos and roots imply that spatial regulation via the corresponding promoter sequences is also essential for metabolic engineering. These results establish a molecular basis for engineering MCFA composition by coordinating the expression of transcriptional regulators such as *OsWRI1a*, and specific thioesterases to accumulate MCFAs with minimal negative agronomic consequences [75].

### 5.1. Role of Lipids in Pollen Fertility of Rice

In rice, for male reproductive organ development the outer lipid layers of the cuticle, which form the outer layer of anther and pollen wall, are important. The anther cuticle consists of cuticle wax and cutin, and exine consists of sporopollenin, which is a more resistant biopolymer made of fatty acid [76]. Under defective lipid synthesis in exine or cuticle tissues, plants are sterile, producing nonviable pollen [77]. One such gene *OsGL1-5*, also known as wax-deficient anther1 (*Wda1*), was cloned from a T-DNA insertional mutant that exhibited pollen sterility and notable abnormalities in the production of very-long-chain fatty acids in two layers [78]. The outermost layer of anther was found to lack epicuticle wax crystals, and the mutant anthers’ ability to generate pollen exine was found to be impaired. In addition to *Wda1*, pollen exine development and fertility are also linked to rice acyl-CoA synthetase5 (*OsACOS5*). Fat alcohol produced by fatty acyl-CoA reductase is a significant fat component found in plant cuticles [79]. One new fatty acid reductase that mediates the synthesis of 1-hexadecanol is called *DPW* (*Defective Pollen Wall*). Mutations in the *DPW* and its *Arabidopsis* homolog *MS2* result irregular exine in anthers and pollen grains, indicating that these genes are functionally conserved [80].

According to a recent study, epigenetic control may affect lipid metabolism. The generation of pollen exines is facilitated by *PEM1* (*pollen-expressed MBD-like 1*), which codes for a methyl-CpG-binding domain protein [81]. Compared with WT plants, *pem1* anthers shrank and produced fewer viable pollen grains, decreasing by 30%. *Pem1* mutants exhibited typical programmed cell death PCD progression of the tapetum, whereas other male sterile mutants typically show a delay in the tapetum breakdown [82]. Additionally, *Pem1* displayed enlarged anther cuticles, delayed exine occurrence, faulty exines, and aberrant Ubisch body development.

The selection of SSR markers associated with humidity-sensitive genic male sterility (*HGMS*) genes would greatly contribute to hybrid rice production. The responsible gene, *HMS1* encodes a potential β-ketoacyl-CoA synthase [83]. *HMS1* prevents pollen from drying up in low air humidity by facilitating the synthesis of C26:0 and C28:0 on the pollen wall that is derived from C24:0 and C26:0 fatty acids by the activity of an *HMS1*-co-factor (*HMS1I*) [84]. *HMS1* and *HMS1I* promote the production of VLCFAs greater than C24 in a yeast system. It is important to note that the researcher used *HMS1* gene to develop a line (*HGMS* line) with ambient pollen fertility across different moisture levels, suggesting that *HMS1* has potential for use in hybrid breeding between *indica* and *japonica* rice. *OsGL1-4/CER1* is involved in male reproductive development and as with other *OsGLs*, in drought stress. *OsGL1-4/CER1* plays a significant role in anther development and plastid differentiation, though it contributes to VLC alkane biosynthesis [85]. A high level of *OsGL1-4/CER1* transcription was observed in bicellular pollen cells and the developing tapetum [86].

### 5.2. Role of Lipids in Rice Grain Yield

Enoyl-CoA hydratase (ECH) catalyzes the second stage of the physiologically significant beta-oxidation pathway in fatty acid metabolism. An enoyl-CoA hydratase/isomerase encoded by the *number of grains 1* (*NOG1*) gene regulates the number of grains per panicle without affecting the number of panicles or grain weight [87]. Twelve-base pair insertion or deletion in the promoter region of *NOG1* affected its transcription, leading to differences in the linolenic acid (LA, C18:3) and total fatty acid content among rice varieties. Since C18:3 is the synthetic precursor of jasmonic acid (JA), *NOG1* contributes to the production of JA biosynthesis and fatty acid metabolism. Grain production increased by up to 25.8% when *NOG1* was introduced into modern cultivars, demonstrating the enormous potential of lipid-related genes in agricultural genetic improvement. A miR1432, a microRNA that is primarily expressed in seeds. In a field trial, suppression of miR1432 increased grain production by up to 17.14% and enhanced grain filling rate [88]. In the fatty acid desaturation and elongation pathway, OsACOT is an essential enzyme, particularly in converting palmitic acid 16:0 into linoleic acid 18:2. An approximate 50% increase in yield was obtained by over-expressing the miR1432-resistant version of *OsACOT*, which mimics miR1432 suppression lines [89].

### 5.3. Regulation of Rice Seed Longevity During Storage

About 3% of rice harvests are lost annually due to ageing during seed storage. A major issue in achieving higher yields and superior quality is the decline in seed germination caused by poor seed storability [90]. Therefore, improving seed storability is essential to ensuring seed safety and preservation under standard storage conditions [91]. In general, environmental and genetic factors affect seed storability during the post-harvest, plant growth, and seed maturity phases. Improving storage conditions is often not economically feasible due to high labor and resource costs. However, improving the storability of rice grains is a highly successful application of genetic traits through breeding programs [92]. More than 70 QTLs related to seed storability have been identified by QTL mapping and association analysis under both artificial and natural aging conditions [93]. Several genes associated with storage resistance have been successfully cloned. These cloned genes include, for instance, the genes *OsLOX1*, *OsLOX2*, *OsLOX3*, and *OsLOX10*, the genes involved in detoxification, *OsALDH7*, *OsGLYI3*, and *OsAKR1*, the genes *OsHSP18.2* and *OsMSRB5* that scavenge reactive oxygen species, the genes OsPIMT1 and PIMT2 for protein repair, the antioxidant enzymes OsCSD1 and OsCSD2 [94]. These discovered genes have greatly improved our understanding of the molecular mechanism underlying seed storage resistance [95].

Alterations in seed lipid composition may affect the utilization of reserves during germination and early seedling growth [96]. Studies with *Arabidopsis* and *Camelina* lines accumulating up to 30–40% MCFAs indicated that normal germination (>95%) was observed when MCFAs were predominantly found in TAG, while their overaccumulation (>50%) in membrane lipids resulted in a minor delay of germination and a lowering of vigor by 5–15% [97]. Although similar studies in rice are scarce, balanced accumulation of MCFAs across different TAGs is an important factor for stable seed physiology. Countermeasures If co-expressed with *DGAT1/2*, oleosin genes may increase oil-body stability193 to reduce any impact; antioxidants (e.g., in the tocopherol pathway) could also improve membrane protection during storage [98]. The inclusion of germination and vigor tests in the MCFA rice breeding program would ensure that MCFA rice does not have a detrimental effect on physiological integrity [99].

### 5.4. Rice Grain Hardness Corelated to Lipid

The milling and texture characteristics of the rice kernel, such as transparency and hardness or softness, are considered as crucial factors in defining the end-use quality of rice products for processing firms and consumers [100]. Lipids present on the surface of starch may bind puroindoline, a protein, to create lipid-protein complexes that play an important role in grain hardness. These surface lipids appear to be more abundant in soft seed kernels than in hard seed kernels [101]. Rice grain hardness is mainly reduced by the expression of puroindoline genes (*pinA* or *pinB*), thereby increasing the percentage of flour particles and starch damage after milling. Cereal kernel hardness was also positively correlated with other endosperm polar lipids, such as LPLs complexed with amylose within starch granules [102]. Chalky endosperm can also arise from changes in the lipid content of rice grains. Endosperm lipids play a role in determining kernel texture, particularly in the hardness and softness [103].

### 5.5. Aroma and Flavor

The aroma and flavour of cooked rice are considered crucial quality attributes that significantly affect palatability and consumer preference [104]. In the rice sector, lipids, particularly the surface FAs, are often regarded as indicators of potential off-flavor and odor development. Volatile substances that interact with olfactory receptors give rice its aroma. 2-AP (2-Acetyl-1-pyrroline) is frequently recognized as a significant aromatic ingredient and has been shown to impart a distinctive aroma to both non-aromatic and uncooked rice [105]. The FAs classes are believed to be responsible for the different types of rice flavour, as several studies have identified that the odor-active compounds among the volatiles are derived from lipids and formed as a result of the chemical degradation of oleic, linoleic, and linolenic acids during storage [106]. Researchers reported that FA is constantly generated during rice milling and then oxidized due to lipases that are remaining on the rice kernel. For instance, compounds such as Octanal, (E)-2-nonenal, heptanal, nonanal, 2-heptanone, and decanal were derived from C18:1, while hexanal, pentanol, pentanal, (E)-2-octenal, (E, E)-2, 4-decadienal, and 2-pentylfuran were often formed from C18:2. The development of off-flavor and the production of rice oxidation volatiles are significantly influenced by a various environmental conditions [107].

### 5.6. Stress Response of Lipids in Rice

Cellular cold resistance is closely associated with the degree of fatty acid saturation in membrane lipids. Research indicates that fatty acid metabolism and membrane lipids significantly influence rice resistance to cold temperatures. Due to the high level of unsaturated fatty acids in the membrane, rice’s resistance to cold is greatly improved, thereby decreasing its phase transition temperature [108]. Acyl carrier protein OsMTACP2 contributes to cold resistance by participating in lipid metabolism, which promotes anther and pollen growth at low temperatures through wax synthesis. Tapetum and pollen grains of anthers are the primary sites of expression of *OsMTACP2*, and mutants lacking this protein have difficulty developing the anther cuticle and pollen wall at low temperatures [109]. Rice leaves and anthers are the main tissues in which the genes *OsKASI-2* and β-ketoacyl-ACP synthase (*KASI*), which encode an indispensable enzyme in lipid biosynthesis, are expressed. KAS enzyme processes, the activity of enzyme KAS, and fatty acid contents decrease in rice due to the loss of function, and the degree of unsaturation of membrane lipids is also affected, making rice more sensitive to chilling stress. Other stress parameters are affected by lipid metabolism and cold acclimation of plants is related to cold tolerance as well [110]. Rice stress tolerance is derived from the rice HTS1 (high temperature sensitive 1) encoding a functional β-ketoacyl carrier protein reductase that regulates fatty acid synthesis and stress signaling pathways. A decrease in fatty acids reduces the stability and integrity of the cell membrane under heat stress, while loss of HTS1 activity directly reduces fatty acid synthesis. *STH1/AOPEPRL1*, encoding an α/β-fold domain-containing hydrolase, is required for rice resistance to salt stress and is functionally involved in plant fatty acid metabolism as a fatty acid hydrolase, and modulates plasma membrane fluidity and integrity due to salt stress [111]. The *OsCDS5* gene encodes CDP-DAG synthase, an enzyme involved in phospholipid synthesis, a structural component of the cell membrane. Up-regulated reactions to biotic and abiotic stress are associated with altered lipid metabolism, driven by altered *OsCDS5* activity [112]. *OsCDS5* mutations boost the expression of several genes involved in defense and promote the generation of reactive oxygen species [113]. Apart from temperature, drought, salinity, and osmotic stress, these factors also have dramatic impacts on rice lipid remodeling. During drought stress, dryness alter membrane lipid profiles, particularly a decrease in levels of phosphatidylglycerol (PG) and phosphatidylcholine (PC) level, which are essential for maintaining cell integrity and avoiding oxidative damage [114]. Enzymes such as phospholipase D (PLD) and lipoxygenase (LOX) are induced to participate in lipid-derived signaling, which promotes drought stress tolerance via ABA-dependent pathways. Likewise, rice plants also dynamically alter the content of UFAs (predominantly mediated by desaturation enzymes FAD2 and FAD8) under salinity stress to maintain membrane fluidity and ion homeostasis [115]. High contents of linolenic acid (C18:3) have been associated with increased salt tolerance, primarily because of its participation as an antioxidant and in signalling. All plant cell membranes are composed of several lipid classes: fatty acids (FAs), glycerolipids, glycerophospholipids, sphingolipids, and galactolipids which function in structure and signaling [116]. Nevertheless, the galactolipid composition of the thylakoid membrane is profoundly altered under saline conditions and a modified ratio (MGDG/DGDG) is associated with different organization and efficiency of the photosynthetic [117]. The higher chlorophyll content, and galactolipid synthesis may be associated with lower salt injury in GA 3-treated plants [118].

Recent studies have shown that simultaneous stresses (such as drought + heat or salinity + temperature) can result in non-additive responses in lipid metabolism. These intricate relationships may result in changes in fatty acid desaturation patterns and TAG accumulation that are differ from those under single-stress conditions [119]. Hence in the next study, we must elucidate the mechanisms by which various abiotic factors affect together, on lipid biosynthetic pathways, which is crucial for developing rice varieties adapted under climate changing [120].

## 6. Genetic Regulation of Fatty Acid Biosynthesis in Rice

An evolutionary split in the regulation of MCFA storage could be demonstrated by comparing FATBs from rice (OsFATB) and coconut (UcFATB). While OsFATB enzymes possess marked substrate specificity for long-chain acyl-ACPs (C16:0–C18:0), leading to the typical LCFA-rich oil of classical rice, UcFATB has evolved a divergent active site harbouring high affinity towards medium-chain acyl-ACPs (C8:0–C12:0) [121]. This basic distinction, due to differences in specific amino acid residues which determine a narrower and more hydrophobic substrate-binding pocket in UcFATB, explains much higher level of natural abundance of MCFAs contained in coconut. The functional implications of this divergence are illustrated by heterologous expression studies in which transfer of UcFATB to rice results in a profound remodelling of lipid profile and carbon partitioning towards MCFA synthesis, thereby substantiating the prospect for harnessing yet another evolutionary advancement to metabolic design [122]. Fatty acid biosynthesis in rice is mainly regulated by a complex gene and transcription factor network that governs the enzymatic steps of fatty acid elongation, desaturation, and modification [94]. MCFA is produced from LCFAs by the action of key enzymes, including medium-chain acyl-CoA synthetase (MCAS), medium-chain acyl-CoA dehydrogenase (MCAD), and other chain-shortening enzymes. They can catalyze the shortening of the fatty acid carbon chain; so, these enzymes are important for increasing MCFA level in rice [123]. Numerous studies have demonstrated that the fatty acid desaturase (FAD) gene family is involved in fatty acid composition in rice. For instance, *FAD2* controls the fatty acid desaturation and thus may affect the chain length of synthesized lipids. However, the subsequent increase in MCFAs has shifted interest towards fatty acid chain-shortening enzymes. Genetic evidence indicates that it is possible to increase the production of shorter-chain fatty acids in rice by overexpressing the medium-chain acyl-CoA synthetase-encoding genes [124]. Quantitative trait loci (QTLs) are specific regions of the genome that contain at least one gene associated with phenotypic variation in complex traits. Although there is abundant genetic diversity in rice subspecies and varieties, lipid and fatty acid composition in rice are generally quantitative traits [125]. Many QTLs for lipid content in rice have been verified in different rice families [56]. One of the QTL that controlling oil content in rice was identified within the specific interval *R1629-XNpb37* on chromosome 10. Rice’s fat level affects its nutritional value, eating quality, and storage stability. It appears that 48 QTLs related to fat content have been identified. More QTLs are found on chromosomes 1, 3, and 6 than on any other chromosome [126]. A study reports 14 QTLs for brown rice crude fat content distributed across chromosomes 1, 3, and 5. One of these is a significant QTL called *qCFC5*, located on chromosome 5 and identified in three populations simultaneously, along with two QTLs on chromosome 7. Major QTLs that are consistently expressed make good candidates for marker-assisted breeding to increase FC. For FC, ten conditional QTL and eleven unconditional QTL were found, with more QTL expressed during the early stages of development [127].

**Figure 4:**
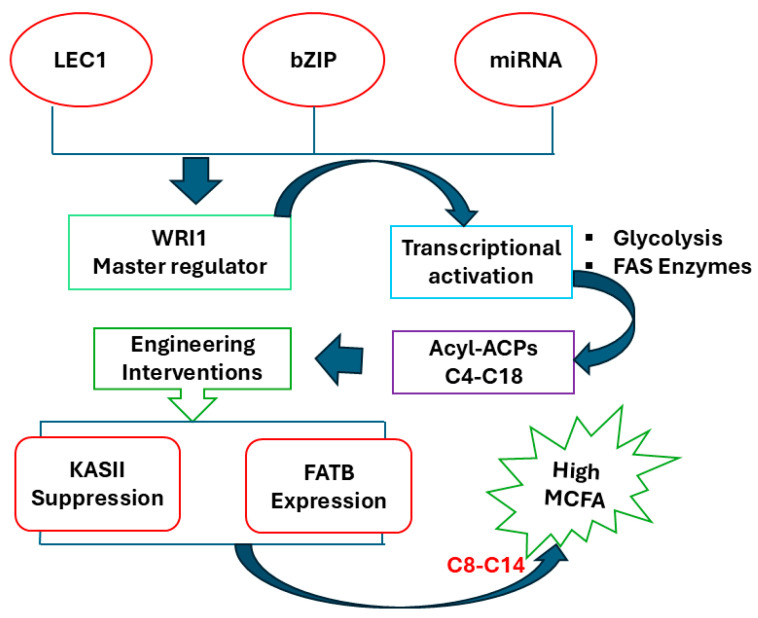
Metabolic pathway design for high-yield MCFA biosynthesis. Increased transcriptional activation (*LEC1*, *bZIP*, and *WRI1*) increases flux into FA synthesis. Selective inhibition of *KASII* and expression of *FATB* leads to carbon flux towards medium-chain fatty acids (C8–C14) instead of long-chain (C16–C18).

There are 29 related QTLs were found across the rice genome, with the except on chromosomes 9 and 10. Eleven mapped QTLs co-localized with nine rice orthologs of *Arabidopsis* genes that encode important lipid metabolism [15]. Rice lipids have thus been recognized as useful dietary molecules. Rice’s lipid content has a relatively high heritability (60.90% to 68.25%) and has been described as a quantitative feature regulated by a polygene [128]. Three QTLs linked to rice’s lipid content were found on chromosomes 1, 2, and 5 [129]. Twelve QTLs were found on chromosomes 1, 3, 4, 5, 7, 8, and 12. Four QTLs governing the lipid content of brown rice were identified on chromosomes 3, 5, 6, and 8 [130]. Many QTLs with major additive effects on the crude FA content of brown rice have been identified by subsequent investigations. Among them, *qLc-5* exhibited the highest effect and was derived from the maternal parent in the study population (Figure 4).

### 6.1. Applications of Genetic Engineering to Increase Fatty Acid

Genetic modification is a potential approach to improve the fatty acid profile of rice, particularly by increasing MCFA synthesis. CRISPR/Cas9 is a robust tool for fine-tuning of FA metabolic genes [131]. For instance, accelerating the gene sequences *ACS* and *MCAS* can accelerate the biosynthesis of medium-chain fatty acids while retaining only the desired features of high-yielding, stress-resistant rice seeds. Recently, the developed CRISPR/Cas9 tool has been used to edit major genes in rice to alter lipid profiles. Using a gene knock-out or gene overexpression system in genes like *FAD2* and *FAD3* can change the ratio of saturated to unsaturated fatty acids in rice, and provide an opportunity to develop a rice variety with a more improved MCFA profile [132]. Genome-editing techniques using TALENs or CRISPR/Cas9 have resulted in several *FAD2* knockout mutants in *Camelina sativa* and soybean (*Glycine max*) [133]. In rice, four *FAD2* genes have been reported and are designated as *OsFAD2-1*, *OsFAD2-2*, *OsFAD2-3*, and *OsFAD2-4*. The Os-BSCTV, KARA408, and BSMV microRNA-resistant constructs were designed to harbour C-terminal truncation, resulting in a non-functional OsFAD2-4 protein compared with other FAD2 proteins. The *OsFAD2-1* gene is often the most abundantly expressed *FAD2* gene in rice seeds [134]. *OsFAD2-2* and *OsFAD2-3* genes are also reported to be expressed only in root tissues based on Rice XPro data. In addition to rice lines overexpressing *MCAS* genes such as those from *Cocos nucifera* (coconut) or *Elaeis guineensis* (oil palm), other plants known to produce oil with higher MCFA content can be used to produce rice oils with higher levels of MCFA [135]. Thus, the transfer of genes between species is a powerful approach that may help achieve the desired fatty acid composition in rice [136].

### 6.2. Rice Breeding and Marker-Assisted Selection (MAS)

Researchers are focusing on applying DNA molecular markers to develop new varieties globally as new genetic technologies advance, such as improving quality and quantity at various plant breeding grounds and developing resilience to biotic and abiotic stressors [137]. Traditional breeding selection is labor-intensive, prone to linkage drag, and relies on phenotypic data selection. Therefore, compared to traditional breeding operations, the use of DNA molecular marker methods is quicker, simpler, and less expensive [138]. Grain yield and quality have improved in several crop plants, including barley, maize, wheat, rice, and pearl millet. Commercially relevant variants were produced through the successful breeding of several critical germplasm lines [139]. The majority of genes utilized in MAS and variety production, including those linked to disease resistance (*Xa23*, *Xa21*, *Xa4*, *Pi l*, *Pi-1*, *Pi-2*, etc.), sterility gene, and date heading, as well as genes associated with grain consistency (*Lgc1*, *Wx*, *Frg*, *badh2*) [140]. Through marker-assisted backcrossing, high amylase content was combined with kernel quality attributes such as weight, width to length ratio, basmati fragrance, and bacterial blight resistance [141].

By providing hybrid and inbred variants, these markers aid in the development of rice in Asian nations, increasing its percentage from 11% to 34% [142]. Molecular markers are important genetic tools for capturing genetic variability and detecting genetic diversity, and breeders use these for obtaining versatile forms. Molecular markers associated with variation that have proven useful for dissecting genome dynamics and improving breeding practice are mapped [143]. Forward-looking MAS is also used to accelerate high-MCFA rice cultivar development other than genetic engineering. In this way, MAS allows for the identification of genetic markers associated with favourable traits, such as MCFAs, thereby avoiding expensive and time-consuming field testing. Upon applying these markers in typical breeding programs, breeders are able to better select genetic lines with high MCFA levels in rice. A large number of genetic loci relevant to lipid metabolism have been identified in rice and can be used for MAS to develop rice with high MCFAs biosynthesis. Some investigators have also linked acyltransferases and desaturases-encoding genes with the modification of rice oil profile, which may serve as tags for improved levels of MCFA [144].

## 7. Traditional Breeding Strategies to Endow Rice with High Levels of MCFA

**Figure 5:**
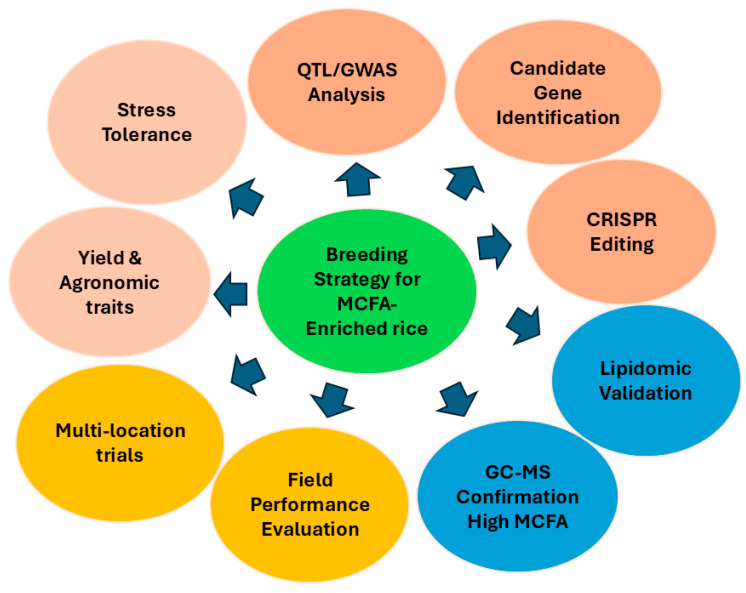
A complete strategy for breeding rice enriched with MCFAs.

Conventional rice breeding has significantly improved agronomic traits, including high yield, quality, and environment resilience. To modify the fatty acid profile, conventional breeding approaches must first screen rice genotypes with natural variation in fat quality. Scientists can also cross rice varieties with higher than normal levels of MCFAs at the beginning of the chain “to stack” the levels of the oils in each generation [145]. There are some drawbacks for traditional breeding/trait improvement of rice in MCFA: (1) genetic variability for MCFA is very low in the rice gene pool, (2) traditional breeding is a lengthy process. As MCFAs are low in most rice cultivars, it will take multiple generations before their levels are appreciably improved [146]. To define conclusive breeding targets, the targets of breeding strategy will be >20% MCFA in seed oil and >22% (*w*/*w*) rice bran oil content [147]. These quantifications are supported by new findings in plant lipid engineering, where related strategies have been successfully applied to increase certain fatty acids in oilseed crops and are further supported by the known natural variation in rice bran composition (Figure 5).

### 7.1. MAS for MCFA Traits in Rice

Marker-assisted selection (MAS), an efficient tool for improving breeding speed, applies the underlying principle of fine-tuning the fatty acid composition in rice breeding. MAS identifies the relevant genetic markers linked to the specific trait of interest, which are used to select individuals with desirable genotypes for breeding programs [148]. There are some QTLs linked to lipid metabolism in rice could be used to select rice lines with elevated MCFA content. Increasing MCFA production, targeted QTLs are associated with acyl-CoA synthetase and fatty acid desaturases [149]. In addition, MAS can assist in identifying genes responsible for stress tolerance and agronomic performance, allowing breeders to select rice varieties with high levels of MCFA while being resistant to environmental stresses such as drought or disease [150].

### 7.2. Phenotyping Methods for the Selection of MCFA-Rich Rice Lines

Efficient phenotyping to screen rice genotypes for a high level of medium-chain fatty acids (MCFA) necessary for breeding. Although these technologies (GC–MS and HPLC) are the gold standard for accurate lipid profiling, they have limited throughput for large breeding populations [151]. New technologies allow for multi-step screening cascades that combine a fast, non-invasive detection step with directed chemical validation. For instance, near-IR and Fourier transform IR (FT) spectral measurements have been calibrated against GC–MS data that gave good predictions of total lipid and the fatty acid chain-length composition in seeds to screen a very large number of samples (thousands) in a short time with more than 85–90% prediction accuracies [152].

Genomic selection (GS) or MAS based on QTLs/SNP markers associated with the *FATB*, *MCAS*, and *FAD2* loci can help reduce the extent of phenotyping required [153]. Furthermore, high-throughput colorimetric or fluorometric assays that detect total acyl-CoA levels and mass pool sampling from early generations can be used as inexpensive preliminary screens prior to detailed lipidomic analysis [154]. Combining these phenomic and molecular tools will provide breeders with the capacity to effectively identify and move MCFA-enriched lines at the population level without sacrificing selection accuracy or resource use [155].

### 7.3. Genetic Engineering and CRISPR/Cas9 for Improved MCFA Content

Genetic modification and gene-editing technologies, including CRISPR/Cas9, are revolutionizing crop breeding by enabling highly targeted modification of genes involved in fatty acid biosynthesis [156]. Scientists can use these advanced tools to edit the genes in rice that already produce MCFA, potentially providing quicker way to develop abundant MCFA maze varieties [157]. *MCAS* gene, one of the central engineers in genetic engineering of rice plants, is responsible for their subsequent activation and production of medium-chain fatty acids [158]. Scientists can transform or overexpress this gene from species have naturally produce high levels of MCFAs, such as coconut or palm kernel, to increase FA production in rice oil. In addition, CRISPR/Cas9 can disrupt or alter the genes responsible for the de novo synthesis of long-chain FAs, causing the metabolism to be switched from the long chain to the medium chain [159]. In addition, compared with traditional transgenic approaches, CRISPR/Cas9-based genetic alterations can be more stable and hence, it is an attractive approach for developing MCFA-enriched rice cultivars [160].

### 7.4. MCFA Improvement Using Transgenic and Hybrid Approaches

Along with CRISPR/Cas9 and gene editing, some transgenic approaches have also specifically transferred foreign genes from other species with MCFA content into rice. For example, rice has been successfully been engineered to contain genes from either coconut (*Cocos nucifera*), or oil palm (*Elaeis guineensis*), producing rice oil with increased levels of fatty acids [161]. Although FA levels in rice can be substantially elevated using these interspecific gene transfer approaches, issues regarding transgene stability and public acceptance of GMOs have hindered their application [162]. Additionally, hybrid breeding approaches that take the best of both worlds, genetically modified and high-MCFA rice that occurs naturally, could provide another potential avenue for crop improvement. Breeders could use MCFA-enriched rice plants generated in the MCFA rice model to cross with elite, high-yield, non-GMO rice varieties to co-develop genotypes with desirable agronomic traits [163]. While various attempts have been made to produce MCFA using natural and recombinant MCFA producers, the accumulation of FA can be toxic to the host, due to intracellular acidification, DNA apurination, protein modification, inhibition of enzymatic activity, and membrane disruption in the microbes [164]. The microbial host sensitivity to the threshold toxicity of MCFA is an important limiting factor that needs to be addressed to achieve high fatty acid production performance. To attenuate MCFA-induced product inhibition due to its toxicity, several process engineering approaches, such as culture broth dilution to reduce MCFA concentration and in situ MCFA extraction, have been proposed [165].

Manipulating the membrane of high fatty acid producers can be a good strategy to protect cells against MCFA and to improve production performance. Another potential approach is to engineer the MCFA transport system to increase the rate of MCFA export from the cell, thereby further strengthening cellular resistance to MCFA inhibition [166]. In research, the export genes of MCFA were characterized by a study of endogenous fatty acid transporters in *E. coli*. By inactivation studies, the *cmr* gene encoding this transporter, annotated to the multidrug efflux superfamily, was shown to mediate MCFA uptake [167]. According to the investigation, the strain of *E. coli.* overexpression of the *acrE*, *mdtE*, *mdtC*, and deletion of *cmr*, were prepared for the combined effect. Consequently, the recombinant strain had a 2 fold higher titer of MCFAs production than the wild-type strain, but its very incomplete cell growth reached the same level as wild-type strain [168]. These results show that modification of the MCFA transport machinery to secrete intracellular MCFA may be a rational approach for improving the viability and MCFA production performance [169]. The introduction or construction of acid resistance systems may be targeted to MCFA producers as an effective approach to developing resistant pathways to MCFA inhibition.

## 8. Challenges in Breeding MCFA-Enriched Rice and Applications

Despite encouraging progress in breeding and genetic engineering of MCFA-enriched rice, there are still several challenges to be addressed. A significant concern is achieving a balance between FA content and rice yield [170]. Elevating the MCFA in the rice fat could deprive the plants from synthesizing the starch or even grain filling, resulting in the plant redirected resources away from seed production and hence reduce the crop yield. Hence, it is important to find a balance between a better MCFA profile and similar agricultural productivity [46]. Changing lipid composition in rice also poses another environmental problem. Alterations in fatty acid composition may in turn, impact the response of the plant to abiotic (e.g., drought, heat) or biotic stress (e.g., pests, pathogens). Evaluate the environmental considerations of GMC their role in plant health and sustainability, whether increasing MCFA is beneficial despite potential negative effects [171].

Because MCFAs are more volatile and chemically unstable than LCFAs, excessive accumulation of MCFA could disturb membrane lipid composition and result in a leaky cellular structure in rice [52]. Increased MCFA reduces the melting points of membrane and affects bilayer dynamics, which can interfere with ion transport, energy balance, and ROS (reactive oxygen species) homeostasis [172]. These unstable structures might accommodate chloroplast and/or mitochondrial malfunction in response to changes in temperature or salinity. Nevertheless, emerging lipidomic and metabolic reports reveal that plants may be able to offset these effects by metabolically sequestering MCFAs in the form of neutral storage lipids (triacylglycerides, TAGs) at the expense of structural phospholipids [173]. Increased activities of acyltransferases such as LPAAT and DGAT induced oleosins and caleosins that protect the membrane phospholipid integrity from lipotoxicity [174]. At the same time, antioxidant networks, such as tocopherols, the ascorbate–glutathione cycle enzymes, and peroxidases, limit the extent of oxidation to maintain redox balance, as demonstrated by recent reports that focusing on oxidative stress regulation via lipid remodelling [175].

The response to abiotic stress can be improved by directing MCFAs into TAGs, as observed in *Arabidopsis* and *Camelina* engineered with *FatB* and *MCM* genes [176]. Therefore, the physiological support for MCFA enrichment in rice involves a concerted lipid partitioning, increased antioxidant defense, and membrane remodeling systems that, together maintain cellular function and metabolism [177].

### 8.1. Health Implications and Nutritional Benefits

Fatty acids have many positive effects on human health, mainly due to their unique metabolic properties. The livers rapidly metabolize the MCFAs from coconut oil to provide the quick source of energy, unlike long-chain fats, which are stored in the body as fat [2]. MCFAs further prevent cognitive decline and have been demonstrated to be therapeutic in neurodegenerative diseases like Alzheimer’s disease. Altering fatty acid composition to increase MCFAs levels provides a promising, healthier alternative to the oils currently used for cooking rice, which primarily contain long-chain fatty acids [178]. These rice oil product types could be a promising base material for the development of functional food products with improved healthful qualities if a high production yield of MCFA-enriched rice oils could be realized [171]. Rice with a better FA composition can assist with weight and blood glucose control and may also decrease the risk of chronic diseases such as cardiovascular disease. In addition to oil modifications, rice starches are also enabling to product development to design specific dietary supplements or therapeutic food products for the management of malnutrition, obesity, and metabolic conditions [179]. Because rice is a staple food in many developing countries, this may be the most cost-effective approach to improving health worldwide [180].

### 8.2. Industrial Applications

Promoting the use of MCFA-rich rice as an energy crop for biofuels could offer an opportunity to mitigate greenhouse gas emissions from agricultural activities and to reduce agriculture’s reliance on fossil fuels [181]. Moreover, since MCFA-rich oils are already being used in various industrial applications, fortifying rice oil with these fatty acids could add a second value for the rice crop as a food-and-fuel crop. They can also be converted to biojet fuel, given their high energy density and renewability as biomass feedstocks for biodiesel production [41]. FAs can serve as unique and supplementary source of natural, plant-derived raw materials for the production of cosmetic formulations, potentially using enriched rice oil [182]. Additionally, MCFAs are used in the pharmaceutical field, as they can serve as useful carriers for drug delivery systems [183]. FAs-enriched oils, such as rice oil, are inexpensive to produce and could be a feasible source for pharmaceutical companies to obtain high concentrations of MCFA-rich oils [184]. Even the food industry benefits from FAs, as they are used to make premium cooking oils, margarine, and spreads. Furthermore, functional foods such as infant formula also contain MCFAs, whose nutritional benefits support brain function and infant growth [185].

### 8.3. Environmental and Sustainability Considerations

Besides the economic and nutritional advantages, enhancing the MCFA content in rice also has sustainability benefits [186]. Increasing the lipid content of rice kernels through genetic engineering could spare the need to grow other, more land- and water-intensive crops, such as palm oil, a commodity closely associated with deforestation and environmental harm [91]. Moreover, to sustain the cultivation of MCFA-enriched rice, sustainable agriculture practices could be adopted, such as decreased water use, organic fertilization, and integrated pest management (IPM), which would help enhance the beneficial effects of MCFA rice, and minimize its environmental impacts. These approaches will ensure that the increased demand for MCFA-enriched rice contributes to and is part of a large set of sustainable agricultural objectives, rather than becoming an environmental issue [187].

### 8.4. Potential Challenges and Pathways Forward

Although MCFA-rich rice has multiple potential applications, a number of hurdles have to be still resolved. The challenge would then be to ensure that increasing the MCFA level would not impair rice yield, disease resistance or stress tolerance [46]. Furthermore, to obtain MCFA-rich rice, significant breeding/genetic modification effort may be required, which may need to be considered on a commercially viable scale, comparing the cost of inputs for genetic engineering or breeding or the added value (and cost of) the MCFA being produced. [188]. The availability and continued improvement of gene-editing tools, transgenic approaches, and marker-assisted selection technologies will be instrumental in developing rice varieties with higher MCFA content without affecting other important agronomic traits [138]. To increase the MCFA content in plants like rice, future work can explore the identification and characterization of genes involved in medium chain fatty acid biosynthesis, including those that regulate the activity of medium-chain acyl-CoA synthetase and the chain-shortening enzyme, to further drive the metabolic pathways responsible for MCFA biosynthesis [189]. Moreover, based on the principle of multi-target, multi-gene engineering could also be a sustainable and efficient pathway for enhancing MCFA accumulation in rice.

### 8.5. MCFA-Enriched Rice in Sustainable Farming Systems

Rice is a major global dietary staple; improving its MCFA content needs to be aligned with sustainable agricultural considerations [190]. Breeding and genetic modification efforts of the future should not only be sustainably oriented but also aim to increase nitrogen-use efficiency, reduce water use, and develop climate-resilient rice varieties [191]. Hence, rice has the potential to provide multiple advantages without any adverse environmental impact if we focus on both increasing the MCFA content and adopting sustainable cultivation methods. In addition, the adoption of MCFA-enriched rice across various cropping systems may yield multiple benefits. Intercropping MCFA-rich rice with other oilseed crops and incorporating it into agroforestry systems could help to restore soil health and biodiversity. This could also help reduce the risks associated with monoculture forms of agriculture and thus contribute to agroecological sustainability [192]. MCFA-enriched rice is currently being applied primarily as healthier edible oils, biofuels, and industrial products, but the possibilities are endless. Focused on the post-harvest significance of MCFA, rice products are now gaining greater global attention and can be produced to meet high market demand including for functional foods, nutraceuticals, and cosmetics [193]. That innovation can greatly meet the demand of sustainable biofuels and that, through the advanced biofuels, MCFA-enriched rice can be a potential feedstock support with clean energy production, surpassing fossil fuels in efficiency. Optimizing the extraction process, alongside future efforts to increase the energy output from MCFA-rich varieties of rice, could make rice an even more promising feedstock for biodiesel and biojet fuel [194].

Although the benefits of MCFA-enriched rice are enormous, it should also be noted that the genetically modified (GM) food crops are often associated with regulatory challenges and public perceptions [195]. Fatty acid-enriched rice GM crops are a rapidly developing field that offers opportunities to address to these problems; however, GM crops are often met with skepticism and resistance during their development, mainly due to safety, ethical, and environmental concerns [196]. Moreover, comprehensive environmental impact assessments will be needed to confirm that the benefits of MCFA-fed rice outweigh any environmental risks. As genetically modified rice offers promising potential for sustainable resource management and improved public health, addressing potential barriers to acceptance through public education and engagement will be crucial [197].

## 9. Future Directions

Metabolic engineering of rice to increase the content of medium-chain fatty acids (MCFAs) offers a paradigm shift that aligns agriculture with nutritional and industrial uses. Unlocking this potential will require a shift from individual modification to systems-level optimization, integrating both basic biological limitations and practical implementation considerations. This integrated approach should utilize the diverse data types from multi-omics studies, genomics, transcriptomics, proteomics, and lipidomics, along with deep haplotype mining of evolving rice pangenome resources to reveal elite alleles and develop predictive models for precision breeding. One of the key challenges is host metabolism, as balanced synthesis of lipids and starches represents an inherent plant trade-off in advanced improved lines. Quantitative analysis reveals that *OsWRI1a* overexpression increases total lipid content by 30–40%, but the average 1000-seed weight is significantly reduced by 15%. Likewise, CRISPR/Cas9-directed mutation of *OsFAD2-1* results in significant endosperm chalkiness (increasing to 25% of the grain area from 5%) and significantly alters the starch viscosity profile, with peak viscosity decreasing by more than 30%. These phenotypes demonstrate the importance of carbon translocation in grain development, yield, and culinary quality. To avoid these trade-offs, elegant genetic engineering strategies are needed to compartmentalize metabolic fluxes. It is essential to restrict the development of MCFAs within non-endosperm tissues; those are particularly specifically active during embryo and bran layers but not starchy endosperm (e.g., using Oleosin and Lipase promoters) and thereby retain the structural and functional integrity with a target MCFA concentration of 20–25% by weight relative to rice oil.

Next-generation editing technologies beyond the CRISPR/Cas9 standard knockouts will have to be implemented in future genetic modification strategies. Innovative technologies, such as CRISPR activation (CRISPRa/dCas9) for multiplexed upregulation of key biosynthetic enzymes, base editing to efficiently install single-nucleotide polymorphisms in regulatory regions, and prime editing for targeted insertions of MCFA-specific thioesterases, provide unprecedented control over the MCFA biosynthesis pathway. These strategies avoid many pleiotropic effects of constitutive overexpression or complete deletion and allow resorts to redirect carbon flow toward MCFA production. At the same time, attention to downstream implications is crucial if successful commercial translation is to be achieved. This involves a detailed investigation of the impact of MCFA enhancement on post-harvest grain processing properties, storage stability against lipid oxidation, and cooking quality parameters. Moreover, it is necessary to take a proactive approach to investigating consumer acceptance through sensory evaluation panels and to have a thorough understanding of the regulations governing the cultivation of genetically modified food crops, direct development towards ready-to-market MCFA-enriched varieties of rice. Target thresholds, such as a minimum 20% MCFA content in seed oil and a grain yield above 90% of conventional varieties, can be set for breeding programs.

Forming a holistic predictive framework encompassing predictive genomics, precise genome engineering, and proactive product characterization, this integrated roadmap provides an unambiguous methodology for navigating the intricate physiological trade-offs. The end goal will be the development of high-yielding, MCFA-rich rice genotypes that realize both opportunities as sustainable sources of improved nutrition and as valuable industrial feedstocks, ultimately contributing to food security and bioeconomic sustainability.

## 10. Conclusions

The development of MCFA-enriched rice is a promising strategy that can supplement rice oil with high-value MCFA, promote the sustainable production of biofuel and serve as a source for industrial products. By utilizing advanced genetic engineering tools, breeding programs, and other biotechnological innovations, scientists can potentially overcome current challenges and enhance rice’s capabilities as a versatile crop. However, despite these advancements, numerous technical, environmental, and societal barriers still hinder the commercialization of MCFA-enriched rice and broader improvements in rice quality, which future research must address. The next generation of sustainable, FAs-enriched rice promises to deliver nutritional benefits and high-value products to agricultural systems, benefiting farmers, consumers, and industries worldwide. Therefore, the commercial cultivation of MCFA-enriched rice will not only improve the nutritional quality of rice but also provide a sustainable solution to meet global energy and industrial needs. With ongoing collaboration among researchers, agricultural practitioners, policymakers, and the general public, MCFA-enriched rice could be a cornerstone of food security, energy sustainability, and healthier diets in the years ahead.

## Figures and Tables

**Table 1:** Key regulatory genes in lipid metabolism.

Gene	Enzyme/Protein	Function on Lipid Metabolism	Expression Sites	Effect on Lipid Profile	References
*OsWRI1*	Transcription Factor	Activates genes for glycolysis and FAS	Seed, Endosperm, Embryo	Increase oil content, Indirect effect on MCFA	[68]
*OsFATB*	Acyl-ACP thioesterase B	Terminates FAS; Release free fatty acid from ACP	Developing seeds, leaves	Increase MCFA and C16:0	[22]
*OsKASII*	β-Ketoacyl-ACP Synthase II	Elongates C16:0-ACP to C18:0-ACP	Developing seeds, Plastids	Potential substrate for MCFA, Increase C16:0 pool	[69]
*OsFAD2*	Fatty acid desaturase 2	Desaturates C18:1 to C18:2	Developing seed, endosperm, roots	Potentially freeing carbon for MCFA synthesis, alter UFA/SFA ratio	[70]
*OsPDAT*	Phospholipid: Diacylglycerol Acyltransferase	TAG synthesis via acyl-CoA-independent pathway	Developing seeds, embryo	Increase the total TAG; unusual FA into storage	[71]
*OsDGAT1*	Diacylglycerol Acytransferase 1	Synthesis of TAG in Kennedy pathway	Developing seeds	Increase TAG, Important for final oil synthesis	[72]

## Data Availability

No new data were generated or analyzed in this study.
